# Deriving Meaning from Chaos: The Mediating Role of the Sense of Coherence in the Serial Relationships among Fear of COVID-19, Indices of Psychological Distress, and Life Satisfaction

**DOI:** 10.3390/healthcare10112276

**Published:** 2022-11-13

**Authors:** Tyrone B. Pretorius, Anita Padmanabhanunni

**Affiliations:** Department of Psychology, University of the Western Cape, Bellville 7530, South Africa

**Keywords:** fear of COVID-19, sense of coherence, hopelessness, anxiety, life satisfaction

## Abstract

The current study focused on the role of the sense of coherence (SOC) in the serial relationships among the fear of COVID-19, indices of psychological distress, and life satisfaction. It examined the hypothesis that an SOC would mitigate the impact of the fear of COVID-19 on psychological distress, which in turn would positively impact life satisfaction. Participants were school teachers (N = 355) who completed the Fear of COVID-19 Scale, the trait scale of the State-Trait Anxiety Inventory, the Beck Hopelessness Scale, the short form of the Sense of Coherence Scale, and the Satisfaction with Life Scale. A path analysis confirmed the mediating role of the dimensions of the SOC in the relationships among the fear of COVID-19, indices of psychological distress, and life satisfaction. Specifically, comprehensibility, meaningfulness, and manageability were found to mediate the associations between the fear of COVID-19 and anxiety, and the fear of COVID-19 and hopelessness, which in turn were associated with higher levels of life satisfaction. The findings confirmed that an SOC is an important source of resilience. Interventions that facilitate the re-appraisal of stressors as challenges and enhance the awareness of practical coping strategies can build an SOC and promote mental health.

## 1. Introduction

The global outbreak of COVID-19, the resultant pandemic, and the related containment and prevention measures have led to a substantive body of research that has highlighted increases in virus-related fear and anxiety [1], mental health problems [2], and social and economic stressors, as well as a decrease in life satisfaction [3]. The fear of contracting COVID-19 can lead to the stigmatization and social exclusion of infected individuals and their families, as well as others associated with the disease (e.g., friends and colleagues). It can also result in heightened levels of anxiety about transmitting the virus to significant others and potentially compromising the safety of loved ones [4]. Elevated rates of mental health problems, such as depression, anxiety, loneliness, and hopelessness, have been documented across diverse settings and among a range of population groups (e.g., university students (Padmanabhanunni and Pretorius [5]), health care workers (García-Fernández and colleagues [6]), and teachers (Baker and colleagues [7])). These studies confirm that the COVID-19 pandemic has posed a significant challenge to individuals’ and communities’ existing coping resources and has led to a loss of a sense of control and stability, increased feelings of helplessness, and reduced life satisfaction [8]. However, not everyone has experienced the same levels of psychological distress. Rather, inherent characteristics or resilience resources have enabled certain individuals to modulate their stress response to the pandemic [3]. Existing research on resilience have conceptualized it as a process of adaptation in the face of significant stress or adversity. Resilience resources confer psychological strength and protect individuals from the potential negative impact of stressors. These resources include social support, adaptive appraisal style, active problem solving, and cognitive flexibility [9,10].

The current study investigated the sense of coherence (SOC) as a resilience resource during the COVID-19 pandemic. The SOC refers to a global and enduring orientation to perceive life and the world as “making sense cognitively, instrumentally and emotionally” [10] (p. 15). The SOC is grounded in the salutogenic theory of health, which offers a paradigmatic shift from the focus on vulnerability factors for adverse outcomes to a search for protective factors that facilitate adaption and coping [10]. The SOC comprises three interrelated components: comprehensibility, manageability, and meaningfulness [11]. Comprehensibility refers to the extent to which the individual perceives the world or external environment as structured, predictable, and consistent, as opposed to chaotic and inexplicable. Manageability reflects the extent to which the individual believes that they have the resources and capacities (e.g., friends, family, religion, financial resources, etc.) to cope effectively with the world. The third component, meaningfulness, is a motivational element that entails the individual’s appraisal that the demands and stressors of life are challenges worthy of emotional investment.

The SOC is not reflective of a particular style of coping. Within the salutogenic framework, the SOC is conceptualized as a trait-like variable that modulates an individual’s position in the continuum between health and pathology [10,11]. Those with a high SOC are better able to negotiate both acute (i.e., sudden and unexpected situations) and chronic stressors (i.e., those that characterize daily life) than their peers by identifying and implementing the most appropriate coping strategy [8]. When exposed to an adverse event, those with a stronger SOC may also experience a decline in mental health but they are more likely to adapt and subsequently achieve a higher level of health following the stressor [10]. A substantial body of research has confirmed that those with a high SOC are less vulnerable to psychological distress than their peers. The SOC has been negatively associated with depression, anxiety, and post-traumatic stress disorder [12]. Furthermore, studies focused on highly demanding situations such as war zones [13] and natural disasters [14] have found that although the SOC weakens in the immediate aftermath of the stressor, individuals with a high SOC tend to recover more rapidly than their peers. It is, therefore, conceivable that the SOC may be an important protective factor in managing distress associated with the COVID-19 pandemic and promoting psychological well-being. Several recent studies conducted during the pandemic [8,15,16] have investigated the SOC as a potential mechanism underlying the relationship between stress and well-being and confirmed its protective role. For example, Barni and colleagues [8] found that the SOC mediated the association between COVID-19 illness experiences (e.g., fear of contracting the virus) and psychological well-being among a large sample of the Italian population. Schäfer and colleagues [16] reported that the SOC predicted changes in psychological distress from pre-pandemic to post-COVID-19 outbreak and that higher levels of the SOC buffered the impact of pandemic-related stressors on mental health. Nevertheless, there remain comparatively fewer studies on the mental health implications of COVID-19 that are grounded in a salutogenic perspective, and our study aims to address this gap in the literature.

The current study aimed to extend the existing research on the role of the SOC in pandemic conditions by examining the role of the three dimensions of the SOC in the serial relationships among the fear of COVID-19, hopelessness, anxiety, and life satisfaction. It was proposed that the dimensions of the SOC mediate the impact of the fear of COVID-19 on hopelessness and anxiety, which in turn leads to greater life satisfaction. Thus, the hypotheses stated below served as a guide for the current study. These hypotheses are grounded in the salutogenic framework, in terms of which a stronger SOC is argued to confer psychological strength. When applied to the COVID-19 context, a higher SOC helps individuals to mobilize resources that can facilitate coping, and this potentially reduces the impact of stressors, lowers negative mental outcomes (e.g., fear, hopelessness, and anxiety), and enhances life satisfaction.

**Hypothesis** **1.** *Comprehensibility mediates the association between the fear of COVID-19 and hopelessness.*

**Hypothesis** **2.** *Manageability mediates the association between the fear of COVID-19 and hopelessness.*

**Hypothesis** **3.** *Meaningfulness mediates the association between the fear of COVID-19 and hopelessness.*

**Hypothesis** **4.** *Comprehensibility mediates the association between the fear of COVID-19 and anxiety.*

**Hypothesis** **5.** *Manageability mediates the association between the fear of COVID-19 and anxiety.*

**Hypothesis** **6.** *Meaningfulness mediates the association between the fear of COVID-19 and anxiety.*

**Hypothesis** **7.** *Comprehensibility mediates the association between the fear of COVID-19 and hopelessness, which is then associated with higher levels of life satisfaction.*

**Hypothesis** **8.** *Manageability mediates the association between the fear of COVID-19 and hopelessness, which is then associated with higher levels of life satisfaction.*

**Hypothesis** **9.** *Meaningfulness mediates the association between the fear of COVID-19 and hopelessness, which is then associated with higher levels of life satisfaction.*

**Hypothesis** **10.** *Comprehensibility mediates the association between the fear of COVID-19 and anxiety, which is then associated with higher levels of life satisfaction.*

**Hypothesis** **11.** *Manageability mediates the association between the fear of COVID-19 and anxiety, which is then associated with higher levels of life satisfaction.*

**Hypothesis** **12.** *Meaningfulness mediates the association between the fear of COVID-19 and anxiety, which is then associated with higher levels of life satisfaction.*

## 2. Materials and Methods

### 2.1. Participants

The sample for this study consisted of a convenience sample of school teachers (N = 355), the majority of whom worked in Western Cape Province in South Africa (82.3%), were women (76.6%), and taught at the primary school level (61.1%). The mean age of the sample was 41.9 years (SD = 12.4). South Africa had 400, 000 teachers in 2021 [17], and our sample of 355 thus represented a 5.09% margin of error with a 95% confidence interval. While we could not perform random sampling due to the lockdown in South Africa, our sample compared favourably with national population data reported by an international survey of teaching and learning [18]. This survey reported that 60% of teachers in South Africa were women, with a mean age of 43 and a mean working experience of 15 years. Chi-square and a one-sample t-test demonstrated that the sample did not differ significantly from the population data statistics. Gender: χ^2^ (1, N = 355) = 0.06, *p* > 0.05. Age: t (354) = 1.68, *p* > 0.05. Teaching experience: t (354) = 1.11, *p* > 0.05.

### 2.2. Instruments

Participants completed the following measures: a brief demographic survey, the Fear of COVID-19 Scale (FCV-19S) [19], the trait scale of the State-Trait Anxiety Inventory (STAI-T) [20], the Beck Hopelessness Scale (BHS) [21], the short form of the Sense of Coherence Scale (SOC-13) [11], and the Satisfaction with Life Scale (SWLS) [22]. The demographic survey focused on participants’ gender, age, and area of residence.

The FCV-19S measures fear reactions toward the COVID-19 pandemic. It consists of seven items to which participants respond on a 5-point Likert scale that ranges from 1 (strongly disagree) to 5 (strongly agree). An example item is: “My hands become clammy when I think about Coronavirus 19.” Several studies have reported satisfactory reliability and validity data in various countries [19,23]. Through a Rasch and Mokken analysis in a South African sample, Pretorius and colleagues [24] demonstrated that the scale is a reliable, valid, and unidimensional measure of the fear of COVID-19.

The STAI-T is a measure of trait anxiety that has been used among healthy and clinical populations [25]. It consists of 20 items assessed on a 4-point scale that ranges from almost never (1) to almost always (4). An example item is: “Some unimportant thoughts run through my mind and bother me.” The evidence for the reliability and validity of the STAI-T is strong, and the scale has been translated into a wide range of languages [25]. In a South African application of the scale, Pretorius and Padmanabhanunni reported a Cronbach’s alpha of 0.90 [26].

The BHS is the most widely used measure of hopelessness. It consists of 20 items that are assessed using a dichotomous true–false response format. An example item is: “I don’t expect to get what I really want.” There is extensive evidence of the reliability and validity of the BHS (see, for example, [27,28]). The BHS was used in a study of South African students that reported a reliability estimate of 0.86 [5].

The SOC-13 measures an individual’s global orientation to life and consists of three subscales: comprehensibility (5 items), manageability (4 items), and meaningfulness (4 items). The SOC-13 is rated on a 7-point Likert scale. Example items include: “Do you have the feeling that you’re being treated unfairly?” (manageability), “Has it happened in the past that you were surprised by the behaviour of people whom you thought you knew?” (comprehensibility), and “Do you have the feeling that you don’t really care about what goes on around you?” (meaningfulness). Manageability, which is considered the instrumental component of the SOC, represents the belief that resources are available to cope with a problem. Comprehensibility, the cognitive component of the SOC, represents the belief that things that happen in life are rational, predictable, and understandable. Meaningfulness, the motivational component of the SOC, represents the belief that things in life are worthwhile. Eriksson and Mittelmark provided a comprehensive overview of the various forms of validity and reliability reported for the SOC-13 and concluded that there is overwhelming evidence that supports the excellent psychometric properties of the scale [29].

The SWLS is the most extensively used measure of satisfaction with life across the world. It consists of five items that are scored on a 7-point Likert scale. An example item is: “The conditions of my life are excellent.” In general, the psychometric properties of the SWLS have been well established (see, for example, [30,31]). The SWLS was used on a sample of South African students, and an alpha coefficient of 0.89 was reported in that study [26]. Classical test theory and item response theory were also used to validate the SWLS in a sample of school teachers in South Africa [32].

### 2.3. Procedure

An electronic version of the instruments was developed using Google Forms. The link was circulated to teachers via social media using Facebook groups that consisted of teachers. In certain instances, the link was emailed on request.

### 2.4. Ethics

The study was conducted according to the guidelines of the Helsinki Declaration. Ethical approval for the study was granted by the Ethics Committee of University of the Western Cape (ethics reference number: HS21/3/8). Participation was voluntary, and participants were required to give informed consent on the first page of the survey before being allowed to proceed to the instruments. They were also provided with contact details for counselling support at the end of the survey.

### 2.5. Data Analysis

**Figure 1:**
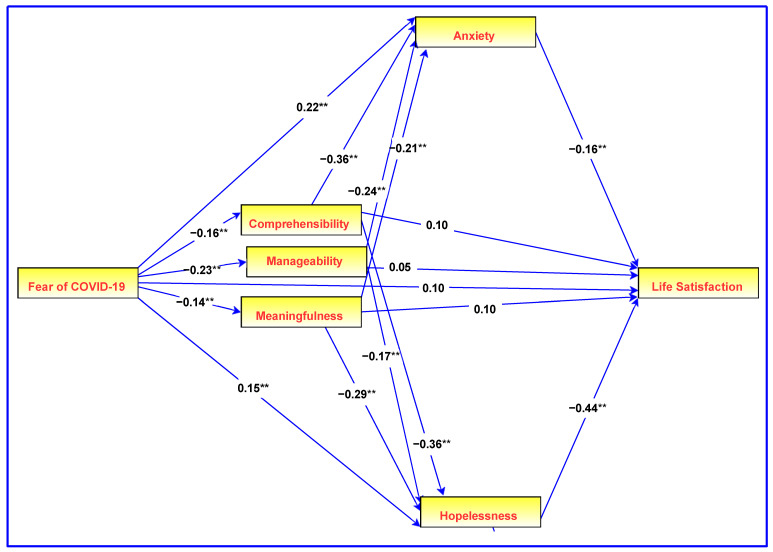
Serial model of the relationships among the fear of COVID-19, the sense of coherence, hopelessness, anxiety, and life satisfaction. Regression coefficients are standardized. ** *p* < 0.01.

Descriptive statistics (means and standard deviations), reliabilities (alpha and omega values), and intercorrelations (Pearson’s correlation) among variables were obtained with IBM SPSS Statistics for Windows (version 26; IBM Corp., Armonk, NY, USA). A path analysis with IBM SPSS Amos (version 26; IBM Corp.) was used to examine the proposed role of the SOC in the serial fear of COVID-19−psychological distress−life satisfaction relationships using maximum likelihood estimation (see Figure 1). Bootstrapped confidence intervals (95%) were used to evaluate statistical significance.

## 3. Results

The descriptive statistics, reliabilities, and intercorrelations of the variables in the study are reported in Table 1. Apart from the meaningfulness subscale of the SOC scale, all the scales demonstrated satisfactory reliability (α and ω = 0.89–0.91). The comprehensibility subscale had moderate alpha reliability (α = 0.69) but satisfactory omega reliability (ω = 0.71), and the manageability subscale demonstrated moderate reliability (α = 0.59, ω = 0.60). The reliability of the meaningfulness subscale was low (α = 0.52, ω = 0.53).

The mean fear of COVID-19 score was 20.9 (SD = 7.1), which was significantly higher than the scores reported for a Spanish sample [33] (M = 16.79, SD = 6.04, t (354) = 10.86, *p* < 0.001) and an Italian sample [34] (M = 16.86, SD = 6.06, t (354) = 10.67, *p* < 0.001). The mean score in the present study was also significantly higher than those found by Winter and colleagues [35] for two different New Zealand samples under differing lockdown conditions (Sample 1: M = 15.6, SD = 7.7, t (354) = 13.99, *p* < 0.001; Sample 2: M = 18.3, SD = 7.9, t (354) = 6.87, *p* < 0.001).

In the current study, the mean score for anxiety was 44.9 (SD = 10.3), which was significantly higher than the scores reported for a South Korean sample [36] (M = 33.1, t (354) = 21.62, *p* < 0.001) and an Italian sample [37] (M = 39.8, SD = 10.4, t (354) = 9.37, *p* < 0.001). It was also significantly higher than those reported by Giner-Bartolome [38] for three different Spanish samples: a healthy control group (M = 18.79, SD = 6.95, t (354) = 47.70, *p* < 0.001), an eating disorder group (M = 33.89, SD = 9.08, t (354) = 20.18, *p* < 0.001), and an eating disorder with self-injury group (M = 39.43, SD = 5.06, t (354) = 10.05, *p* < 0.001).

The mean hopelessness score in the current study was 5.7 (SD = 4.9), which was significantly higher than the scores reported by Kliem and colleagues [39] for a German sample (M = 4.87, SD = 4.33, t (354) = 3.29, *p* = 0.001) and those reported by Yesilcinar and colleagues [40] for a Turkish sample (M = 4.92, SD = 4.02, t (354) = 3.10, *p* = 0.002). It was also significantly higher than the scores reported by İzci and colleagues [41] for breast cancer patients (M = 4.80, SD = 3.62, t (354) = 3.56, *p* < 0.001).

The mean life satisfaction score in the current study was 21.9 (SD = 7.3), which was significantly lower than the scores reported for a Spanish sample [42] 2019: M = 27.1, SD = 5.9, t (354) = −13.45, *p* < 0.001), a sample of women with depressive disorder in Poland [43] (M = 23.9, t (354) = −5.19, *p* < 0.001), and a sample of Colombian women [44] (M = 23.09, SD = 6.9, t (354) = −3.09, *p* = 0.002).

The fear of COVID-19 was positively related to anxiety (r (353) = 0.33, *p* < 0.001) and hopelessness (r (353) = 0.25, *p* < 0.001) and negatively related to the dimensions of the SOC (comprehensibility: r (353) = −0.16, *p* = 0.002; manageability: r (353) = −0.23, *p* < 0.001; meaningfulness: r (353) = −0.14, *p* = 0.010) and life satisfaction (r (353) = −0.11, *p* = 0.04). These findings indicated that high levels of the fear of COVID-19 were associated with high levels of anxiety and hopelessness and low levels of the sense of coherence and life satisfaction. Hopelessness and anxiety were negatively associated with comprehensibility (hopelessness: r (353) = −0.48, *p* < 0.001; anxiety: r (353) = −0.59, *p* < 0.001), manageability (hopelessness: r (353) = −0.47, *p* < 0.001; anxiety: r (353) = −0.57, *p* < 0.001), meaningfulness (hopelessness: r (353) = −0.45, *p* < 0.001; anxiety: r (353) = −0.45, *p* < 0.001), and life satisfaction (hopelessness: r (353) = −0.62, *p* < 0.001; anxiety: r (353) = −0.52, *p* < 0.001). These findings indicated that high levels of the SOC were associated with low levels of hopelessness and anxiety, whereas high levels of hopelessness and anxiety were associated with low levels of life satisfaction. Finally, life satisfaction was positively associated with comprehensibility (r (353) = 0.45, *p* < 0.001), manageability (r (353) = 0.42, *p* < 0.001) and meaningfulness (r (353) = 0.40, *p* < 0.001), which indicated that high levels of the SOC were associated with high levels of life satisfaction.

The path analysis model that was used to examine the direct and indirect effects of fear of COVID-19, together with the standardized regression coefficients, is presented in Figure 1. This model includes the fear of COVID-19 as a predictor, the dimensions of the SOC as mediators of the fear of COVID-19−psychological distress relationship, and life satisfaction as the outcome variable. There were no gender differences in the outcome variable, namely, life satisfaction (t = 1.87, *p* = 0.06); therefore, gender was not added as a covariate in the path analysis model.

The direct effects resulting from the path analysis model are presented in Table 2. The zero-order correlations reported in Table 1 indicated that the fear of COVID-19 was positively related to life satisfaction; however, this relationship was found to be non-significant (β = 0.096, *p* = 0.08, 95% CI [0.00, 0.18]) when considered within the serial model. This finding supported the hypothesis that fear of COVID-19 indirectly impacted life satisfaction (see indirect effects). Similarly, the relationship between the dimensions of the SOC and life satisfaction was significant in the zero-order correlations but non-significant within the serial model (comprehensibility: β = 0.103, *p* = 0.21, 95% CI [−0.03, 0.24]; manageability: β = 0.052, *p* = 0.49, 95% CI [−0.07, 0.18]; meaningfulness: β = 0.092, *p* = 0.09, 95% CI [0.00, 0.18]). This finding indicated that the dimensions of the SOC indirectly affected life satisfaction via the indices of psychological distress.

The indirect effects resulting from the path analysis model are reported in Table 3. The obtained results support all the stated hypotheses. In particular:

1. Comprehensibility mediated the association between the fear of COVID-19 and hopelessness (β = 0.041, *p* < 0.001, 95% CI [0.01, 0.05]).
2. Manageability mediated the association between the fear of COVID-19 and hopelessness (β = 0.039, *p* = 0.007, 95% CI [0.01, 0.05]).
3. Meaningfulness mediated the association between the fear of COVID-19 and hopelessness (β = 0.040, *p* = 0.005, 96% CI [0.01, 0.05]).
4. Comprehensibility mediated the association between the fear of COVID-19 and anxiety (β = 0.058, *p* < 0.001, 95% CI [0.04, 0.13]).
5. Manageability mediated the association between the fear of COVID-19 and anxiety (β = 0.055, *p* < 0.001, 95% CI [0.04, 0.12]).
6. Meaningfulness mediated the association between the fear of COVID-19 and anxiety (β = 0.029, *p* = 0.005, 95% CI [0.01, 0.07]).
7. Comprehensibility mediated the association between the fear of COVID-19 and hopelessness, which in turn was associated with higher levels of life satisfaction (β = 0.041, *p* < 0.001, 95% CI [−0.04, −0.01]).
8. Manageability mediated the association between the fear of COVID-19 and hopelessness, which in turn was associated with higher levels of life satisfaction (β = 0.039, *p* = 0.006, 95% CI [−0.03, −0.01]).
9. Meaningfulness mediated the association between the fear of COVID-19 and hopelessness, which in turn was associated with higher levels of life satisfaction (β = 0.040, *p* = 0.005, 95% CI [−0.03, −0.01]).
10. Comprehensibility mediated the association between the fear of COVID-19 and anxiety, which in turn was associated with higher levels of life satisfaction (β = 0.058, *p* = 0.002, 95% CI [−0.02, −0.004]).
11. Manageability mediated the association between the fear of COVID-19 and anxiety, which in turn was associated with higher levels of life satisfaction (β = 0.055, *p* = 0.002, 95% CI [−0.02, −0.003]).
12. Meaningfulness mediated the association between the fear of COVID-19 and anxiety, which in turn was associated with higher levels of life satisfaction (β = 0.029, *p* = 0.004, 95% CI [−0.01, −0.001]).

## 4. Discussion

The aim of the current study was to extend prior research on the buffering role of the SOC by investigating the role of the three dimensions of the SOC in the relationships among the fear of COVID-19, hopelessness, anxiety, and life satisfaction in a sample of South African school teachers. There were several important findings. First, the study participants’ fears of COVID-19 and the levels of anxiety and hopelessness were higher than those reported for other samples [33,36,40]. Additionally, the levels of life satisfaction were significantly lower in the current sample than those reported in the literature (see, for example, [44]). These findings could be ascribed to the schooling context in South Africa at the time of the study. Data collection was undertaken during the third wave of the pandemic, when conventional classroom teaching was reinstated by the government. Approximately 2283 teachers died from contracting the virus during the first and second waves of the pandemic [45]. It is probable that the awareness of the increased risk of infection heightened the levels of fear and anxiety among teachers. Furthermore, a significant proportion of public schools in South Africa have overcrowded classrooms, poor sanitation, and little access to running water, which could enhance teachers’ sense of vulnerability and fear about contracting the virus [46]. Given this context, the government mandate to resume conventional teaching may have contributed to teachers’ feelings of hopelessness regarding their ability to protect themselves, which in turn may have led to reduced life satisfaction.

Second, comprehensibility, manageability, and meaningfulness respectively mediated the associations between the fear of COVID-19 and hopelessness, and the fear of COVID-19 and anxiety. According to social–cognitive theories (see, for example, Lazarus and Folkman [47]), the extent of psychological distress experienced in relation to a particular stressful situation is determined by the individual’s context-dependent appraisal of that stressor. The outcome of this evaluation influences the individual’s behavioural reaction and motivation to address the stressor. The Positive Appraisal Style Theory of Resilience [48] suggests that protective factors are activated when stressful situations are strong enough to automatically trigger negative appraisals. In these situations, a strong SOC is likely to lead to a more positive re-appraisal of the situation, thereby influencing mental health outcomes [3]. Applied to the current study, the teachers’ SOC could potentially lead them to re-appraise their fear of COVID-19 as an adaptive and reasonable reaction, mobilize resources to manage the situation, and adopt protective measures (e.g., hand hygiene and the proper wearing of face masks). In turn, these actions could promote a sense of stability and confidence in their coping abilities, thereby promoting their well-being.

Third, the three dimensions of the SOC mediated the association between the fear of COVID-19 and hopelessness and the association between the fear of COVID-19 and anxiety, both of which in turn were associated with higher levels of life satisfaction. Life satisfaction refers to an individual’s cognitive evaluations of their life. Teachers in the present study may have appraised the resumption of conventional schooling as necessary in a context in which most students do not have access to online modes of learning. As a result, they may have viewed their work as more meaningful and worthwhile in pandemic circumstances than otherwise [46], which could have impacted their levels of distress and life satisfaction.

The findings of the study have implications for interventions, particularly due to the indication that the SOC may be an important protective factor in reducing the risk of adverse mental health outcomes. The SOC is a skill that can be learned [11]. Supporting individuals in re-appraising stressful events as challenges and enhancing their awareness of practical strategies that can be implemented to enhance coping can promote an SOC [8]. For example, the teachers in the study who re-appraised the return to conventional teaching as an opportunity to demonstrate their commitment and care for the education of students may have enhanced their SOC. Furthermore, official state-sponsored initiatives to acknowledge teachers and demonstrate appreciation for their role, as well as efforts to increase teachers’ access to personal protective equipment, could help to increase teachers’ SOC.

The study had certain limitations. A cross-sectional design was used, which limited the extent to which causal inferences could be made. A longitudinal approach may be beneficial to investigate the long-term influence of the SOC on psychological well-being during prolonged pandemic conditions. An electronic survey was used, which likely limited the study sample to a portion of the population that was more likely to engage with online platforms. Additionally, the results of the study should be interpreted with caution due to the low reliability of the meaningfulness domain of the SOC-13. The study focused on a distinctive subgroup of the population and future research among diverse samples would be beneficial in generating further insights on the role of the SOC in facilitating coping. Despite these limitations, the study confirmed the potential buffering role of the SOC as an importance source of coping and resilience.

## 5. Conclusions

The findings confirm that the SOC is an important protective factor that can modulate mental health outcomes in the context of the COVID-19 pandemic. The study lends further support to salutogenic theory and the importance of interventions aimed at fostering the SOC by facilitating the re-appraisal of stressful situations as worthy of engagement, which can help to build a sense of confidence in one’s coping abilities. Cognitive behavioural interventions that focus on addressing maladaptive appraisals and promoting cognitive flexibility may enhance people’s capacity to cope with stressors related to the pandemic.

## Figures and Tables

**Table 1:** Descriptive statistics, intercorrelations, and reliabilities of study variables.

	1	2	3	4	5	6	7
1. Fear of COVID-19	—						
2. Anxiety	0.33 ***	—					
3. Hopelessness	0.25 ***	0.62 ***	—				
4. Comprehensibility	−0.16 **	−0.59 ***	−0.48 ***	—			
5. Manageability	−0.23 ***	−0.57 ***	−0.47 ***	0.71 ***	—		
6. Meaningfulness	−0.14 **	−0.45 ***	−0.45 ***	0.43 ***	0.42 ***	—	
7. Life satisfaction	−0.11 *	−0.52 ***	−0.62 ***	0.45 ***	0.42 ***	0.40 ***	—
Mean	20.9	44.9	5.7	20.8	15.3	19.8	21.9
SD	7.1	10.3	4.9	5.4	4.4	4.1	7.3
Alpha	0.91	0.91	0.89	0.69	0.59	0.52	0.90
Omega	0.91	0.91	0.89	0.71	0.60	0.53	0.90

*** *p* < 0.001, ** *p* < 0.01, * *p* < 0.05.

**Table 2:** Direct effects of predictors within the serial model.

Effect	Beta	SE	β	95% CI	*p*
Fear of COVID-19 → Hopelessness	0.097	0.032	0.150	[0.070, 0.230]	0.002
Fear of COVID-19 → Anxiety	0.289	0.062	0.220	[0.148, 0.294]	0.001
Fear of COVID-19 → Meaningfulness	−0.079	0.029	−0.137	[−0.212, −0.048]	0.009
Fear of COVID-19 → Manageability	−0.144	0.032	−0.231	[−0.314, −0.147]	0.001
Fear of COVID-19 → Comprehensibility	−0.125	0.038	−0.164	[−0.248, −0.082]	0.001
Fear of COVID-19 → Life satisfaction	0.090	0.051	0.096	[0.005, 0.182]	0.082
Hopelessness → Life satisfaction	−0.640	0.082	−0.439	[−0.526, −0.345]	0.001
Anxiety → Life satisfaction	−0.113	0.045	−0.157	[−0.262, −0.052]	0.006
Meaningfulness → Hopelessness	−0.324	0.061	−0.291	[−0.378, −0.194]	0.001
Manageability → Hopelessness	−0.173	0.071	−0.168	[−0.279, −0.060]	0.010
Comprehensibility → Hopelessness	−0.211	0.055	−0.249	[−0.358, −0.141]	0.001
Meaningfulness → Anxiety	−0.474	0.111	−0.209	[−0.290, −0.129]	0.001
Manageability → Anxiety	−0.503	0.134	−0.239	[−0.344, −0.136]	0.001
Comprehensibility → Anxiety	−0.614	0.106	−0.355	[−0.452, −0.260]	0.001
Meaningfulness → Life satisfaction	0.149	0.089	0.092	[0.002, 0.183]	0.092
Manageability → Life satisfaction	0.079	0.115	0.052	[−0.069, 0.179]	0.493
Comprehensibility → Life satisfaction	0.127	0.103	0.103	[−0.034, 0.240]	0.208

Note. Beta = unstandardized coefficient, β = standardized coefficient.

**Table 3:** Indirect effects of fear of COVID-19 on hopelessness, anxiety, and life satisfaction.

Effect	Beta	SE	β	95% CI	*p*
Fear of COVID-19 → Comprehensibility → Hopelessness ^1^	0.026	0.011	0.041	[0.012, 0.050]	0.000
Fear of COVID-19 → Manageability → Hopelessness ^2^	0.025	0.012	0.039	[0.009, 0.049]	0.007
Fear of COVID-19 → Meaningfulness → Hopelessness ^3^	0.026	0.011	0.040	[0.010, 0.045]	0.005
Fear of COVID-19 → Comprehensibility → Anxiety ^4^	0.077	0.028	0.058	[0.039, 0.134]	0.000
Fear of COVID-19 → Manageability → Anxiety ^5^	0.073	0.024	0.055	[0.039, 0.123]	0.000
Fear of COVID-19 → Meaningfulness → Anxiety ^6^	0.038	0.017	0.029	[0.014, 0.069]	0.005
Fear of COVID-19 → Comprehensibility → Hopelessness → Life satisfaction ^7^	−0.017	0.008	0.041	[−0.035, −0.007]	0.000
Fear of COVID-19 → Manageability → Hopelessness → Life satisfaction ^8^	−0.016	0.008	0.039	[−0.033, −0.006]	0.006
Fear of COVID-19 → Meaningfulness → Hopelessness → Life satisfaction ^9^	−0.016	0.007	0.040	[−0.031, −0.006]	0.005
Fear of COVID-19 → Comprehensibility → Anxiety → Life satisfaction ^10^	−0.009	0.004	0.058	[−0.021, −0.004]	0.002
Fear of COVID-19 → Manageability → Anxiety → Life satisfaction ^11^	−0.008	0.005	0.055	[−0.019, −0.003]	0.002
Fear of COVID-19 → Meaningfulness → Anxiety → Life satisfaction ^12^	−0.004	0.003	0.029	[−0.010, −0.001]	0.004

Superscript numbers 1–12 correspond to the numbering of the hypotheses.

## Data Availability

The data that support the findings of this study are available from the corresponding author, upon reasonable request.
